# Generation-based life table analysis reveals manifold effects of inbreeding on the population fitness in *Plutella xylostella*

**DOI:** 10.1038/srep12749

**Published:** 2015-07-31

**Authors:** Lu Peng, Mingmin Zou, Nana Ren, Miao Xie, Liette Vasseur, Yifan Yang, Weiyi He, Guang Yang, Geoff M. Gurr, Youming Hou, Shijun You, Minsheng You

**Affiliations:** 1 Institute of Applied Ecology, Fujian Agriculture and Forestry University, Fuzhou 350002, China; 2 Fujian-Taiwan Joint Centre for Ecological Control of Crop Pests, Fujian Agriculture and Forestry University, Fuzhou 350002, China; 3 Key Laboratory of Integrated Pest Management for Fujian-Taiwan Crops, Ministry of Agriculture, Fuzhou 350002, China; 4 Department of Biological Sciences, Brock University, St. Catharines, Ontario L2S 3A1, Canada; 5 Graham Centre, Charles Sturt University, Orange, New South Wales 2800, Australia; 6 Department of Botany, University of British Columbia, 3529-6270 University Blvd. Vancouver, BC V6T 1Z4, Canada

## Abstract

Understanding how inbreeding affects fitness is biologically important for conservation and pest management. Despite being a worldwide pest of many economically important cruciferous crops, the influence of inbreeding on diamondback moth, *Plutella xylostella* (L.), populations is currently unknown. Using age-stage-specific life tables, we quantified the inbreeding effects on fitness-related traits and demographic parameters of *P. xylostella*. Egg hatching rate, survival and fecundity of the inbred line significantly declined compared to those of the outbred line over time. The inbred *P. xylostella* line showed significantly lower intrinsic rate of increase (*r*), net reproduction rate (*R*_0_), and finite increase rate (*λ*), and increasing generation time (*T*). Inbreeding effects vary with developmental stages and the fitness-related traits can be profoundly affected by the duration of inbreeding. Our work provides a foundation for further studies on molecular and genetic bases of the inbreeding depression for *P. xylostella*.

Inbreeding is recognized to increase homozygosity and generate genetically less diverse offspring^1^. Inbreeding results in adverse effects on life-history traits in many species^2^ and a reduction in fitness^3^,^4^, called inbreeding depression.

Inbreeding depression has been well studied in insects^5^, covering a variety of morphological, physiological and life-history traits^6^, including egg hatching^7^,^8^, larval survival^9^, overwintering survival^10^, developmental time or growth rate^11^, developmental stability (e.g., fluctuating asymmetry^12^), female fecundity^13^, male fertility^14^, adult mating behavior, sperm competition or mating success^8^,^15^, and adult lifespan^9^. The deleterious effects can greatly vary among populations within a given species or among species^1^. To date, however, how inbreeding affects population demography has been fairly limited, especially when it is based on evaluation of age-stage-specific traits, which is important for metamorphic insects^16^.

The life table approach is commonly employed to study how the demography of an insect population may change in response to environmental variables^17^,^18^. It has been a useful tool to detect inbreeding in the mosquito, *Culex tritaeniorhynchus*^19^ for example. Demographic parameters, which incorporate life-history information of a population as values, such as intrinsic rate of increase (*r*), finite rate of increase (*λ*), net reproductive rate (*R*_0_) and mean generation time (*T*), are useful estimators to predict the potential growth of the subject population^20^.

The diamondback moth, *Plutella xylostella*, (Lepidoptera, Plutellidae) is a cosmopolitan, and oligophagous pest of cruciferous plants^21^. Because of the abundance of cultivated and weedy host plants, widespread resistance to insecticides, limited natural enemy impact as a consequence of insecticide use, and high fecundity^22^, it has become one of the most widely distributed lepidopteran pest species in the world^23^. We first observed the deleterious effects of inbreeding in *P. xylostella* when using an inbred population for sequencing its genome^24^,^25^. To date, however, no study has examined how inbreeding affects life history traits and population demography of such a serious pest insect worldwide.

This study was designed to quantify the manifold effects of inbreeding on the population fitness of *P. xylostella*. We used the age-stage and two-sex life table approach^26^ to examine the life-history traits including survivorship, development and fecundity, and evaluate its impacts on the population demography of different generations in the *P. xylostella* outbred and inbred colonies. The results of our study showed that the life history traits and demographic parameters were influenced by the history of inbreeding, and the inbreeding effects were developmental stage and age specific. These findings provide an important basis for further studies to uncover molecular insights of inbreeding depression of *P. xylostella* using a genomic approach.

## Results

### Age-stage specific survival rate

**Figure 1:**
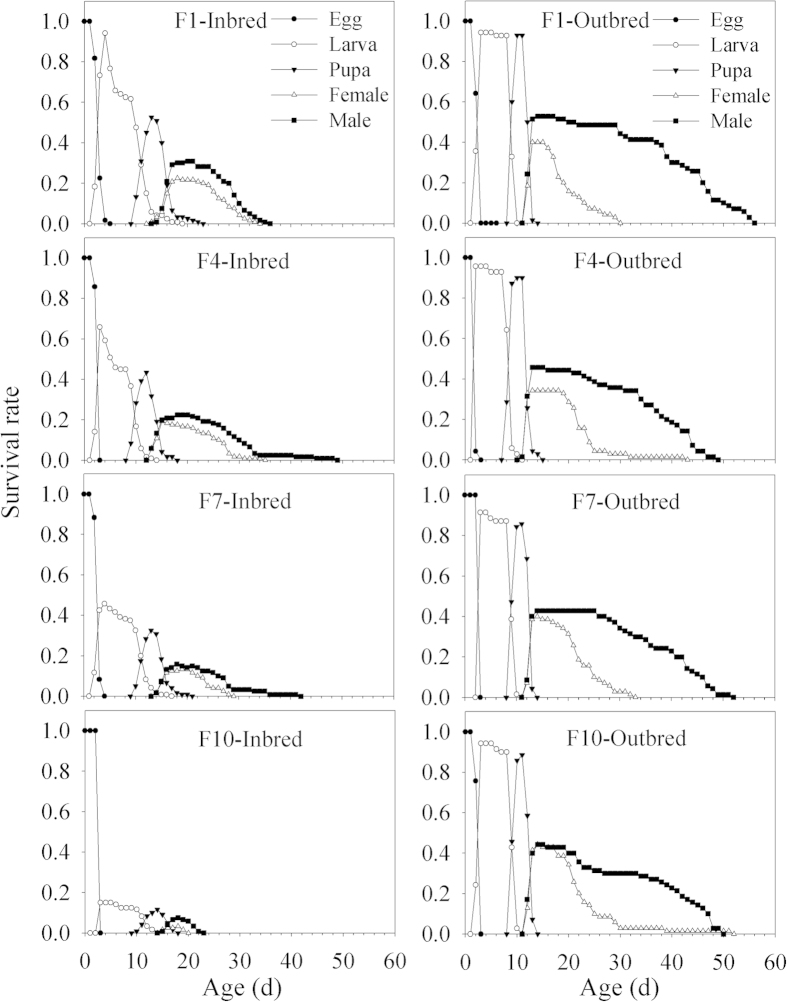
Age-stage specific survival rates (*s*_*xj*_) of different generations (F 1, 4, 7 and 10) in the inbred and outbred lines of *P. xylostella*.

The age-stage survival curve, *s*_*xj*_, depicts the probability that a newly laid egg will survive to age *x* and stage *j*. The overlaps between different stages occurred as a result of the developmental differentiation among individuals. The outbred line showed a similar pattern of projected curves for each developmental stage. For example, the peaks of egg-hatching curves varied from 0.91 to 0.96 with an average of 0.94 ± 0.01. The peaks of the curves for pre-adult survival rates (i.e. the probability of a newly laid egg surviving to adult stage) for the four presented generations varied from 0.83 to 0.93 with an average of 0.88 ± 0.02 (Fig. 1).

Through the four presented generations, the proportion of individuals in each developmental stage varied to a greater extent in the inbred line than the outbred line (Fig. 1). This is reflected by a progressive decline in the peak of hatching success rates, from 0.97 in F1 to 0.67 in F4, 0.53 in F7, and then 0.15 in F10 and pre-adult survival rates, from 0.55 in F1 to 0.12 in F10. In the outbred line, hatching success rate (0.91 ~ 0.96) and pre-adult survival rate (0.83 ~ 0.93) remained relatively constant over the four presented generations (Fig. 1).

**Figure 2:**
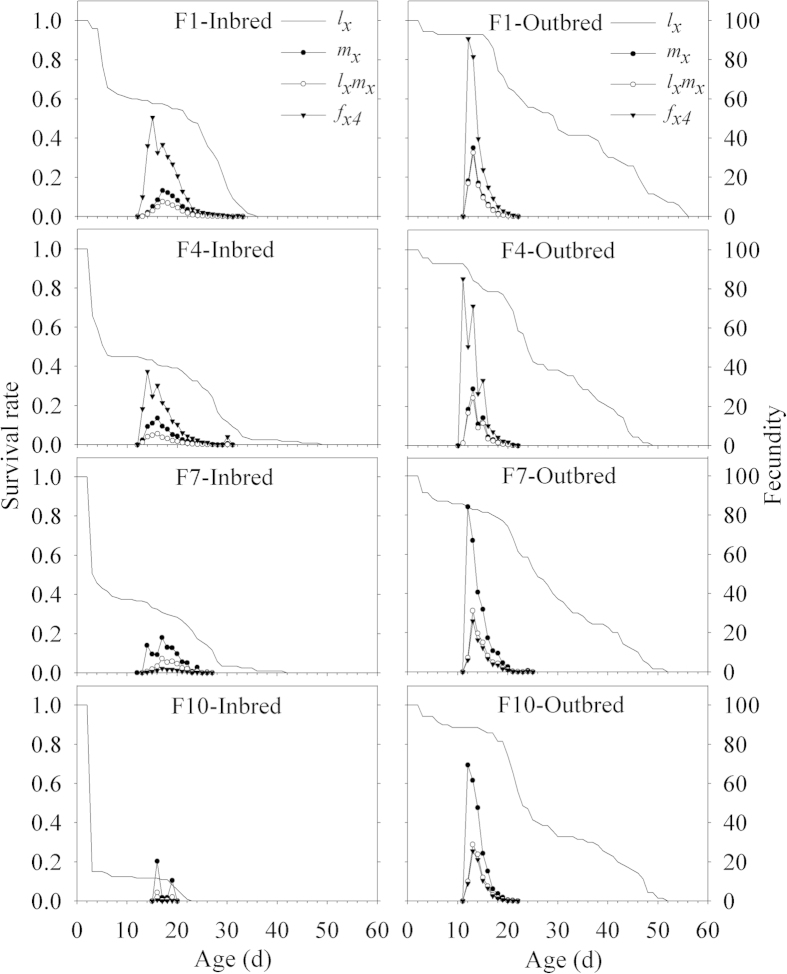
Age-specific survival rate (*l*_*x*_), age-specific fecundity (*m*_*x*_), age-specific maternity (*l*_*x*_*m*_*x*_), and age-stage specific adult female fecundity (*f*_*x4*_) of different generations (F 1, 4, 7 and 10) in the inbred and outbred lines of *P. xylostella*.

By ignoring the stage differentiation, a single age-specific survival rate (*l*_*x*_) gives the probability that an egg will survive to age *x*, and we found that in the inbred line, *l*_*x*_ on day 3 went from 96% in the F1 to 15% in the F10 (Fig. 2) while the *l*_*x*_ of the outbred line was relatively constant over the four presented generations. A similar pattern of the probability to survive to day *x* was observed for all generations in the outbred line with usually half of the individuals surviving longer than 23 d (Fig. 2).

### Age-stage specific fecundity

The number of offspring produced by an individual *P. xylostella* of age *x* and stage *j* is shown in Fig. 2. Because only females produce eggs, there is only a single curve (*f*_*x4*_) that represents the females (stage 4). No different patterns of the dynamics of the *f*_*x4*_, *m*_*x*_, and *l*_*x*_*m*_*x*_ were observed among the four outbred generations, while they all showed a declining trend over different generations in the inbred line (Fig. 2). The peaks of *f*_*x4*_ in each of the four presented generations of the outbred line were much higher than that in the inbred line. This value tended to sharply decrease with increasing inbred generations. Maximum *f*_*x4*_ was reached on average from the 12^th^ to 14^th^ day in the outbred line while it ranged from the 14^th^ to 18^th^ day in the inbred line, showing that the inbreeding delayed peak oviposition in addition to depressing overall fecundity. The curve for *m*_*x*_ was lower than the *f*_*x4*_ in inbred and outbred lines because it was a parameter of age-specific averaged fecundity taking into account of concurrent stages.

### Development, longevity, and fecundity

The generalized linear model with repeated measures showed that the stage-specific developmental durations of *P. xylostella* were significantly different between the two breeding lines (*χ*^2 ^= 52.90, df = 1, *P* < 0.001 for egg; *χ*^2 ^= 217.38, df = 1, *P *< 0.001 for larva; *χ*^2 ^=173.26, df = 1, *P *< 0.001 for pupa; and *χ*^2 ^= 467.61, df = 1, *P *< 0.001 for pre-adult), being significantly prolonged in the inbred line (Table 1). In general, there were significant differences in stage-specific developmental durations among generations for all stages (χ^2 ^= 102.65, df = 3, *P *< 0.001 for egg; χ^2 ^= 23.06, df = 3, *P *< 0.001 for larva; χ^2 ^= 12.00, df = 3, *P *< 0.001 for pupa; and χ^2 ^= 93.02, df = 3, *P *< 0.001 for pre-adult). However, these differences did not show any upward or downward trends either within the outbred or the inbred line, suggesting that they varied randomly (Table 1).

There were no significant differences in longevity of adult females between the two breeding lines (*χ*^2 ^= 4.41, df = 1, *P *= 0.120) and among generations (*χ*^2 ^= 4.73, df = 3, *P *= 0.193). However, further post-hoc sequential Bonferroni tests showed that the inbred F10 (3.5 ± 0.6 d) was significant shorter than the outbred line F10 (11.3 ± 1.3 d) and other inbred generations (Table 2), suggesting that the inbreeding effects might be closely related to the inbreeding history. The longevity of adult males was significantly different between the two breeding lines (χ^2 ^= 130.03, df = 1, *P *< 0.001) as well as among generations (*χ*^2 ^= 14.41, df = 3, *P *= 0.002), with the inbred line being significantly shorter than the outbred line in each generation and fell to a minimum of 4.3 ± 0.6 d in the inbred F10. However, no significant differences were observed among different generations in the outbred line as well as among the first three generations in the inbred line (Table 2). There was a sharp decline of longevity in the inbred F10 suggesting impact of inbreeding.

Mean fecundity per female significantly differed between the two breeding lines (χ^2 ^= 47.38, df = 1, *P *< 0.001) and among generations (χ^2 ^= 35.28, df = 3, *P *< 0.001) (Table 2). Mean fecundity per female in the outbred line ranged from 177.7 ± 11.4 to 213.2 ± 8.1 eggs and did not vary significantly over the different generations, according to post-hoc sequential Bonferroni test (Table 2). In the inbred line, fecundity did not differ between F1 (180.9 ± 12.8) and F4 (143.3 ± 11.6). However, with increasing number of the inbred generations, fecundity dropped significantly to 76.4 ± 5.7 in F7, and to 23.5 ± 1.3 in F10 (Table 2). The significant differences occurred only in F7 and F10 generations, consistent with a cumulative effect over successive generations.

### Age-stage specific life expectancy

**Figure 3:**
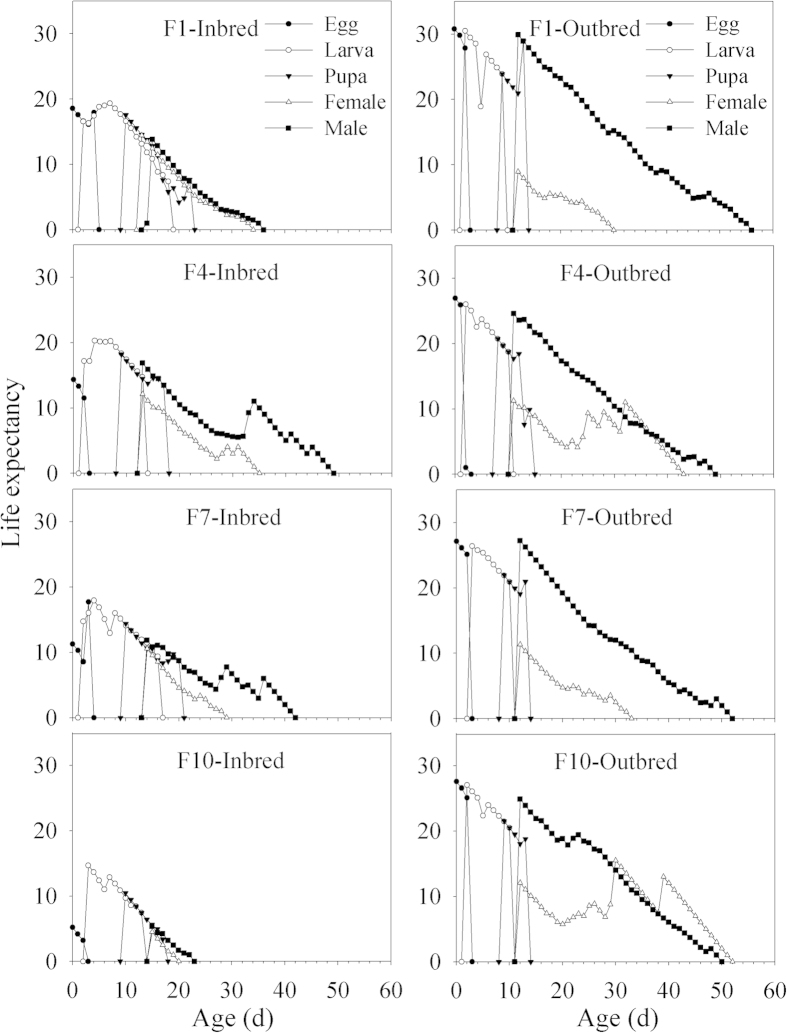
Age-stage- and sex-specific life expectancy (*e*_*xj*_) of different generations (F 1, 4, 7 and 10) in the inbred and outbred lines of *P. xylostella*.

The *e*_*xj*_ gives the expected life span that an individual of age *x* and stage *j* can live after age *x*. Generally, *e*_*xj*_ was higher for the outbred line than the inbred line (Fig. 3). For example, *e*_*01*_ in the outbred line were 30.8, 26.9, 27.1 and 27.6 d in F1, F4, F7, and F10, respectively, which were markedly higher than those in the inbred line (18.6, 14.3, 11.3 and 5.2 d). Within the inbred line, *e*_*xj*_ gradually decreased over successive generations.

### Age-stage specific reproductive value

**Figure 4:**
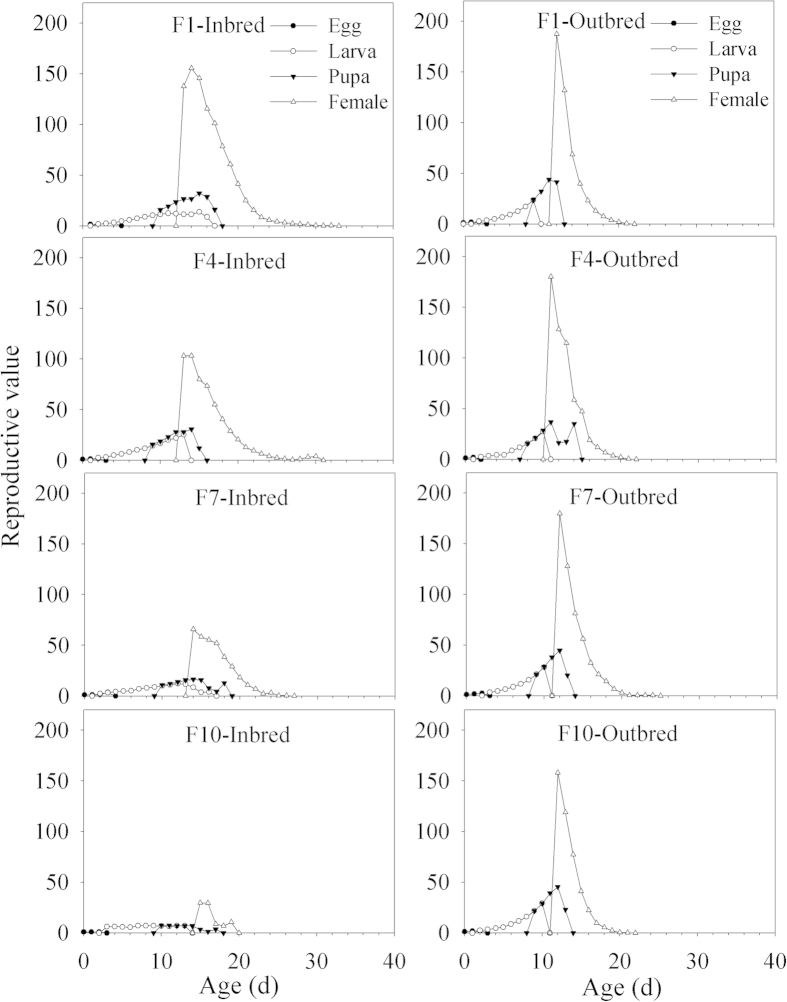
Age-stage reproductive value (*v*_*xj*_) of different generations (F 1, 4, 7 and 10) in the inbred and outbred lines of *P. xylostella*.

The *v*_*xj*_ is the contribution of individuals of age *x* and stage *j* to the future population. The outbred line maintained relatively constant *v*_*xj*_ over the different generations (Fig. 4). The *v*_*xj*_ of the inbred line in each generation was lower than that of the outbred line. Peak *v*_*xj*_ occurred at ages 14 d (155.4 eggs), 13 d (103.2 eggs), 14 d (65.7 eggs), and 15 d (29.7 eggs) for F1, F4, F7, and F10, respectively, in the inbred line. These were later, as well as conspicuously lower than those of the outbred line occurring at ages 12 d (187.4 eggs), 11 d (180.2 eggs), 12 d (179.8 eggs), and 12 d (158.0 eggs) for F1, F4, F7, and F10, respectively. The values of *v*_*xj*_ were found to gradually decline with successive generations, and the highest values in F10 (29.7 eggs) was observed being about 15% of F1 (155.4 eggs).

### Population demographic parameters

The means and standard errors of the population demographic parameters estimated employing the bootstrap techniques are listed in Table 3. The intrinsic rate of increase (*r*), finite rate of increase (*λ*), and net reproduction rate (*R*_0_) in the outbred line were consistently and significantly higher than those in the inbred line (*P *< 0.05) for all generations. In the outbred line, these parameters (*r*, *λ*, *R*_0_) did not differ significantly among the four generations (*P *> 0.05). In the inbred line, however, *r* significantly decreased in F7 with a sharp decline to 0.0109 in F10 (*P*<0.05). A similar downward trend was observed for *λ* in the inbred line, showing an increase of the inbreeding effect mostly after F4. *R*_0_ steadily decreased from 36.4 in F1 afterward to 1.3 in F10 (*P *< 0.05).

The generation time (*T*) was significantly prolonged in the inbred line (*P *< 0.05) compared to the outbred line and these differences were significant in all successive generations. However these differences also did not show any significant trends in either line, suggesting random variation (Table 3).

## Discussion

The lower and decreasing egg-hatching rate of the inbred *P. xylostella* line compared to that of the outbred line provides further evidence to support the documented negative effects of inbreeding on egg hatching in insects^6^,^7^,^8^. Such intergenerational effects of inbreeding may be explained by the fact that early development is controlled by maternally derived proteins and mRNA transcripts in the fertilized eggs, which depend on maternal genotype and thus maternal inbreeding status^6^. Further, inbred male insects have been found to produce fewer and poorer quality sperm^27^. This leads inbred females to have fewer fertilized eggs, thus reducing their fecundity and possibly producing more unhealthy eggs than in outbred line^6^.

The decline in survival rate of pre-adults further demonstrated the fact that eggs produced by sib mating pairs were less likely to survive to the adult stage than those from outbred line. These results are consistent with other studies^28^,^29^. Nepoux *et al*.^30^ report that moderate inbreeding over two generations in *Drosophila* is sufficient to reduce egg-to-adult viability. It is known that inbred organisms are often more susceptible to environmental challenges^31^,^32^. This may explain to some extent the lower survival rate at the larval stages of the inbred *P. xylostella* as a result of the weak endurance to environment, although conditions were maintained optimal and constant in our study.

Reproduction remains one of the most important determinants of population fitness, especially in insect species that typically produce most offspring at an early age and have no parental care^7^. Examples include *Callosobruchus chinensis*^7^, *Cylas formicarius elegantulus*^33^, and *D. melanogaster*^34^. Some studies argue that lower metabolic efficiency in homozygous individual may play a key role in explaining inbreeding depression^35^, with genetic stress leading to less energy allocated to reproduction^36^,^37^. This may help interpret our observation of a lower fecundity of the inbred *P. xylostella* compared to that of outbred line. Interestingly, sexual dimorphism in lifespan could result from sex-specific selection, caused by fundamental differences in how males and females optimize their fitness by allocating resources into current and future reproduction^37^,^38^,^39^. In our study, male longevity of the inbred line was significantly shorter than that of the outbred line over successive generations, suggesting a gradually increasing effect of inbreeding on the lifespan of *P. xylostella* adult males as previously reported in *D. melanogaster*^37^,^40^. The assumption is that the optimal reproductive strategies of males and females differ, causing that limited energy resource availability must be balanced between longevity and successful mating of males (e.g., search for mating, competition and courtship)^38^,^39^,^41^. Therefore, we speculate that the reduction in male longevity of the inbred *P. xylostella* might attribute to fitness costs or trade-offs of the energy required for mating behaviors^31^,^42^.

Our study showed that the impact of inbreeding on the developmental time for pre-adult stages was less profound than that on the survival rate and fecundity for *P. xylostella*. Fox and Scheibly^43^ indicate that inbred beetle *Stator limbatus* takes only 1.5 days longer to reach the adult stage than outbred line, while egg hatching rate and larval survival are both significantly suppressed in the inbred line. Wright *et al*.^42^ also report more significant effects of inbreeding on the longevity and fecundity of adults than that on the developmental time in *D. simulans*. Similarly, low to negligible effects of inbreeding on the developmental time have been reported for *Gryllus firmus*^11^ and *D. melanogaster*^34^. The effect of inbreeding on a given trait depends upon the proportion of directional dominance on that trait^1^. This directional dominance indicates that life-history traits can be differentially affected by inbreeding, depending on the link of those traits with fitness and the proportion of directional dominance that exist among those traits^5^. This may help explain a lower level of effect of inbreeding on developmental time than on survivorship and fecundity in *P. xylostella*.

Environmental variables can affect the magnitude of inbreeding^34^,^44^. Our study was performed in the laboratory under optimal conditions for growth and development of *P. xylostella* and might somewhat mask the inbreeding effect^15^. Thus, the resulting fitness of inbred insects including *P. xylostella* could be even greater or lower in the field. On one hand, *P. xylostella* is a specialist herbivore and food availability may contribute to inbreeding pressures. Temporal and spatial variation of Brassica species at the landscape level may lead *P. xylostella* to exist as metapopulations with common local extinction events. Such a structure might reduce genetic diversity and maintain inbreeding depression over time, inhibiting the capacity of a species to colonize and adapt to novel habitats^46^,^47^. On the other hand, *P. xylostella* evolves to colonize and adapt to diverse environments worldwide^23^ and gene flow can be quite high among population helping to maintain genetic variability in populations^32^. Morse^48^ suggests that the low inbreeding effects on *S. limbatus* survival and development may be related to its dispersal activities in nature, which may promote gene flow and thus limiting inbreeding depression in wild populations^47^,^49^,^50^. How *P. xylostella* evolves to colonize and adapt to diverse environments worldwide remains unknown and requires further studies to reveal genetic and molecular mechanisms for its success.

In summary, using the age-stage-specific life table approach, our study reveals that inbreeding can have deleterious effects on the population fitness-related traits of *P. xylostella*. Specifically, we found that population traits such as rates of egg hatching, survivorship and fecundity, as well as on the population demographic parameters (*r*, *R*_0_ and *λ*) were significantly reduced in the inbred line. The observed patterns of the fitness traits in the inbred line were profoundly influenced by the history of inbreeding over different generations, suggesting the importance of genetic load in inbreeding depression. Here, we performed the experiment using a laboratory population with relatively identical genetic background, which might mask the inbreeding effect in outdoor populations of *P. xylostella* as reported in butterflies^15^. The generation-related variation in the fitness traits provides a solid foundation to further examine the factors that govern the genetic load, and test the assumption of the mutation–selection model^5^,^14^,^51^. In the present study, therefore, we aimed to quantify how the fitness-related traits vary with different generations of inbreeding using the life table approach. To better understand the genetic load of inbreeding depression and the function of key genes associated with population fitness of *P. xylostella*, further studies are required to confirm our results either by using different outdoor populations or using a molecular approach, such as specific regulatory genes and their regulation pathways involved in the deleterious effects, based on the available genomic data^25^.

## Materials and Methods

### *P. xylostella* strain rearing

To ensure relatively identical genetic background of *P. xylostella*, we used an insecticide susceptible strain (Fuzhou-S) as the initial population, which was collected from a cabbage (*Brassica oleracea* var. *capitata*) field in Fuzhou (26.08°N, 119.28°E) in 2004 and used for genome sequencing^25^. This original colony was reared on potted radish seedlings (*Raphanus sativus*) at 25 ± 1 °C, 65 ± 5% RH and L:D = 6:8 h in a separate greenhouse without exposure to insecticides for the past eight years, and its susceptibility to insecticides (fipronil and chlorpyrifos) was tested and confirmed in our laboratory^52^. In the inbreeding experiment, insects were reared on five-to-seven-true-leaf stage cabbage plants, *B. oleracea* var. *capitata* (Jing Feng-1) at 25 ± 1 °C, 65 ± 5% RH and L:D = 16:8 h in a separate greenhouse.

### Inbred and outbred lines

**Figure 5:**
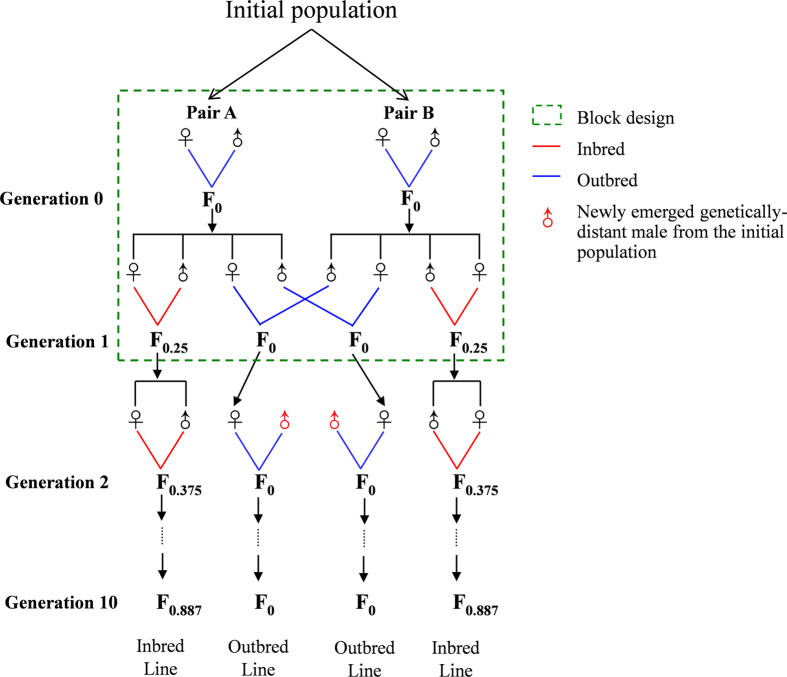
Crossing design for generating serial inbred and outbred lines of *P. xylostella*.

Prior to the inbreeding experiment, the initial population was kept under random mating to maintain high genetic diversity. The inbreeding experiment started using a “block” design (Fig. 5)^53^. This design enables to minimize errors in sampling alleles while creating lines, and ensure the same effective population sizes at the beginning of the experiment for inbred and outbred lines^53^. To start the experiment, the block was created with two pairs of *P. xylostella* adults (Pair A and B in Fig. 5) sampled randomly from the initial population. The offspring from each of the pairs were regarded as generation 0 (F0), and we randomly chose two female and two male offspring to start serially inbred and outbred (control) lines.

For the serially inbred line, in each generation, one virgin female was paired to her full brother for sibmating. The eggs produced by the pair were collected, grown to adulthood and again one male and female collected to use to start the next generation (see next paragraph for the detailed procedure used in this experiment). This was repeated for 10 generations. For the serially outbred line, at each generation, one virgin female was randomly paired with a newly emerged genetically-distant male from the initial population, except for F1 that was produced from F0 (Fig. 5), to ensure their outbred nature^13^. While individual life history traits and demographic parameters were measured in each generation, we present here the analyses using generations 1, 4, 7, and 10.

The effects of sib mating on the development and survival of offspring were tested at each generation by first collecting the newly-laid eggs and rearing them in incubators (MHT350, Sanyo Electric, Osaka, Japan) running at 25 ± 1 °C, 65 ± 5% RH and L:D = 16:8 h. The newly emerged adults were then separated by sex and one male and one female were paired in the plastic cups (9.5 cm in top diameter, 5 cm in bottom diameter, 15 cm in height) to produce the next generation, as described previously. The bottom of each cup was removed and the base covered with a fine mesh gauze, which was then inverted and placed over a plastic Petri dish (9 cm in diameter) prior to introduction of the moths. A cotton wick soaked with 10% honey solution was provided as food. A fresh cabbage leaf with moist cotton ball wrapped around the petiole was placed inside each of the cups for egg laying. The cabbage leaves with eggs laid within a 24 h period were individually transferred into plastic Petri dishes with a moistened filter paper for the subsequent examination. Given that the mortality might be high in the inbred line (due to inbreeding depression), 120 fresh eggs were used as initial population of each generation for the age-stage-specific life table studies of the inbred line.

For the outbred line, a similar procedure was applied however, instead of 120 eggs, 70 fresh eggs were used to start each generation. The hatching rate of eggs for each generation of inbred and outbred lines were examined and recorded daily. Moist filter papers were regularly changed to maintain humidity. Each of the hatched larvae was moved into a new plastic Petri dish as previously described. Fresh cabbage leaves and moist filter papers were changed daily until the larvae pupated. Developmental time and daily survival rate of each larva were recorded. Pupae were collected and kept individually in glass tubes (1 cm in diameter, 4 cm in height) for emergence and sex determination.

Once adults emerged, they were paired and subsequently kept in individual plastic cups with 10% honey soaked cotton wick as food. Each of the adult pairs was moved daily into a new cup with a fresh cabbage leaf. Daily monitoring was performed to record the number of eggs laid and the longevity of adults for each of the populations.

### Demographic data analyses

Data obtained from the inbreeding experiments were analyzed using an age-stage and two-sex life table approach^26^. The life history parameters, including age-stage specific survival rate (*s*_*xj*_) (where *x* is age and *j* stage (egg, larva, pupa and adult)), age-specific survival rate (*l*_*x*_), age-stage specific fecundity (*f*_*xj*_), age-specific fecundity (*m*_*x*_), age-specific maternity (*l*_*x*_*m*_*x*_), age-stage specific life expectancy (*e*_*xj*_), reproductive value (*v*_*xj*_) and the demographic parameters of intrinsic rate of increase (*r*); finite rate of increase (*λ*); net reproductive rate (*R*_0_) and mean generation time (*T*) were estimated using Chi and Liu’s^26^ methodology. Analyses of the raw data and calculation of life history parameters were performed with TWOSEX-MSChart^54^ which is designed in Visual BASIC for Windows operating system and available at http://nhsbig.inhs.uiuc.edu/wes/chi.html (Illinois Natural History Survey, Champaign-Urbana, IL).

### Statistical analyses

Since data coming from each of the lines (inbred or outbred) were not independent and did not meet normality assumption, a generalized linear model (GLM) with linear distribution was used to compare the developmental duration, fecundity, and adult longevity among generations (within subject factors) and between inbred and outbred lines (between subject factors). This approach is considered appropriate for autocorrelated data and is frequently used when the assumptions for analysis of variance (ANOVA) may be violated^55^. Analyses used SPSS 17.0.

For population demographic variables, the bootstrap^56^ technique included in the TWOSEX-MSChart was used to estimate the means, standard errors and variances of the population parameters with 10,000 permutations. Within the TWOSEX-MSChart, the Tukey–Kramer tests and two-sample *t* tests^57^,^58^ were used to compare difference in population parameters among generations and between inbred and outbred lines within one generation.

## Additional Information

**How to cite this article**: Peng, L. *et al*. Generation-based life table analysis reveals manifold effects of inbreeding on the population fitness in *Plutella xylostella*. *Sci. Rep*. **5**, 12749; doi: 10.1038/srep12749 (2015).

## Figures and Tables

**Table 1:** Generation-based stage-specific developmental duration (day) for inbred and outbred lines of *P. xylostella*.

Gen.	Egg		Larva		Pupa		Pre-adult		
	Outbred	Inbred	Outbred	Inbred	Outbred	Inbred	Outbred	Inbred	
F1	2.6 ± 0.1Ab	3.1 ± 0.1Bb	6.7 ± 0.1Aa	8.7 ± 0.2Bb	3.2 ± 0.1Aa	4.6 ± 0.1Bc	12.6 ± 0.1Aab	16.2 ± 0.2Bb	
F4	2.0 ± 0.0Aa	2.8 ± 0.0Ba	6.8 ± 0.1Aa	7.6 ± 0.1Ba	3.5 ± 0.1Ab	4.0 ± 0.1Bab	12.3 ± 0.1Aa	14.3 ± 0.2Ba	
F7	3.0 ± 0.0Ac	2.9 ± 0.1Aab	6.5 ± 0.1Aa	8.9 ± 0.2Bb	3.4 ± 0.1Aab	3.9 ± 0.1Ba	12.9 ± 0.1Ab	15.8 ± 0.2Bb	
F10	2.7 ± 0.1Ab	3.0 ± 0.1Aab	6.8 ± 0.1Aa	9.0 ± 0.3Bb	3.2 ± 0.1Aa	4.2 ± 0.2Bbc	12.7 ± 0.1Aab	16.4 ± 0.3Bb	

A generalized linear model with repeated measures with a post hoc sequential Bonferonni test was used to determine the significant differences between lines and among generations within each line. The capital letters show significant differences between breeding lines in each generation, while the small letters indicate the significant differences among generations within each line (*P *< 0.05).

**Table 2:** Generation-based adult longevity and female fecundity for inbred and outbred lines of *P. xylostella*.

Gen.	Female longevity (day)		Male longevity (day)		Female fecundity (eggs/female)	
	Outbred	Inbred	Outbred	Inbred	Outbred	Inbred
F1	8.4 ± 0.8Aa	11.5 ± 0.7Bb	29.4 ± 1.6Aa	12.3 ± 0.7Bb	213.2 ± 8.1Aa	180.9 ± 12.8Aa
F4	10.2 ± 1.1Aa	10.9 ± 1.1Ab	23.7 ± 1.7Aa	15.5 ± 1.3Bb	178.0 ± 17.0Aa	143.3 ± 11.6Aa
F7	10.5 ± 0.8Aa	8.9 ± 0.6Ab	26.4 ± 1.4Aa	10.8 ± 1.2Bb	192.7 ± 13.6Aa	76.4 ± 5.7Bb
F10	11.3 ± 1.3Aa	3.5 ± 0.6Ba	24.2 ± 2.0Aa	4.3 ± 0.6Ba	177.7 ± 11.4Aa	23.5 ± 1.3Bc

A generalized linear model with repeated measures with a post hoc sequential Bonferonni test was used to determine the significant differences between lines and among generations within each line. The capital letters show significant differences between breeding lines in each generation, while the small letters indicate the significant differences among generations within each line (*P *< 0.05).

**Table 3:** Generation-based population parameters for inbred and outbred lines of *P. xylostella*.

	Intrinsic rate of increase (*r*)		Finite rate (*λ*)		Net reproduction rate (*R*_0_)		Generation time (*T*)	
	Outbred	Inbred	Outbred	Inbred	Outbred	Inbred	Outbred	Inbred
F1	0.308 ± 0.011Aa	0.193 ± 0.012Ba	1.361 ± 0.015Aa	1.213 ± 0.015Ba	85.4 ± 12.8Aa	36.4 ± 7.0Ba	14.4 ± 0.1Aa	18.5 ± 0.4Ba
F4	0.297 ± 0.013Aa	0.172 ± 0.015Ba	1.345 ± 0.018Aa	1.188 ± 0.018Ba	71.2 ± 12.4Aa	22.9 ± 5.6Bb	14.3 ± 0.1Aa	18.0 ± 0.3Ba
F7	0.289 ± 0.012Aa	0.125 ± 0.017Bb	1.336 ± 0.015Aa	1.133 ± 0.020Bb	77.0 ± 12.6Aa	12.3 ± 3.8Bc	15.0 ± 0.1Ac	19.7 ± 0.5Bb
F10	0.295 ± 0.010Aa	0.011 ± 0.021Bc	1.344 ± 0.014Aa	0.984 ± 0.016Bc	77.7 ± 11.0Aa	1.3 ± 0.4Bd	14.7 ± 0.1Ab	18.0 ± 0.4Ba

following the means  ±  *SE* within the same row in each population parameter indicates significant difference between two crosses (*t*-test; *P *< 0.05).

No identical lowercase letter following the means ±*SE* within the same column indicates significant difference between generations (Tukey-Kramer; *P *< 0.05).
